# Determining SARS-CoV-2 non-infectivity state–A brief overview

**DOI:** 10.3389/fpubh.2022.934242

**Published:** 2022-08-12

**Authors:** Siggeir F. Brynjolfsson, Hildur Sigurgrimsdottir, Olafur Gudlaugsson, Mar Kristjansson, Karl G. Kristinsson, Bjorn R. Ludviksson

**Affiliations:** ^1^Department of Immunology, Landspitali—The National University Hospital of Iceland, Reykjavik, Iceland; ^2^Department of Medicine, Faculty of Medicine, University of Iceland, Reykjavik, Iceland; ^3^Department of Infectious Diseases, Landspitali—The National University Hospital of Iceland, Reykjavik, Iceland; ^4^Department of Clinical Microbiology, Landspitali—The National University Hospital of Iceland, Reykjavik, Iceland

**Keywords:** COVID-19, SARS-CoV-2, transmission of SARS-CoV-2, non-infectious state, viral testing

## Abstract

From the beginning of the COVID-19 pandemic, it has claimed over 6 million lives, and globally the pandemic rages with detrimental consequences, with the emergence of new more infectious and possibly virulent variants. A clinical obstacle in this battle has been to determine when an infected individual has reached a non-infectious state. Severe Acute Respiratory Syndrome Coronavirus 2 (SARS-CoV-2) can be transmitted under diverse circumstances, and various rules and regulations, along with different testing methods, have been applied in an attempt to confine the transmission. However, that has proven to be a difficult task. In this review, we take together recently published data on infectivity and transmission of SARS-CoV-2 and have combined it with the clinical experience that physicians in Iceland have accumulated from the pandemic. In addition, we suggest guidelines for determining when patients with COVID-19 reach a non-infectious state based on a combination of clinical experience, scientific data, and proficient use of available tests. This review has addressed some of the questions regarding contagiousness and immunity against SARS-CoV-2.

## Initial infectious state

The average incubation period for SARS-CoV-2 is 2–7 days, with over 98% of symptomatic patients falling ill within 12 days (1–4). The different variants of SARS-CoV-2 have different mean incubation times, the omicron variant (B.1.1.529) has a mean incubation period of 3.2 days as compared to 4.4 days for the delta variant (B.1.617.2) (5). The delta variant also has a shorter mean incubation period as compared to previous variants (6). The most common way to diagnose COVID-19 infection is by using reverse transcription polymerase chain reaction (RT-PCR) on samples collected from the upper respiratory tract (nasopharyngeal and/or oropharyngeal swabs). SARS-CoV-2 virus in infected individuals can be detected by RT-PCR for an average of 17.0 days in samples taken from the upper respiratory tract, with the highest levels in the first week (7). The mean duration of viral shedding is variable depending on what type of sample is being measured. Viral shedding can be detected by RT-PCR in samples from the lower respiratory tract for an average of 14.6 days while samples from stool are positive for an average of 17.2 days (7).

In some cases, patients can remain SARS-CoV-2 positive by RT-PCR for a prolonged time, up to 230 days in an immunocompromised patient (8) but also in previously healthy individuals for 60–110 days (9–11). However, it is unclear, in these cases, whether the cause of prolonged viral shedding is the retention of the virus in the body or re-infection. It has been reported that patients with COVID-19 can be tested negative after disease followed by a positive test (re-detectable positive) both with the same and different variants or even co-infected by multiple variants of the virus (12, 13). Many individuals test positive for COVID-19 without showing symptoms, after diagnosis, many patients develop symptoms, but some remain asymptomatic. The reported percentage of individuals that remain asymptomatic throughout the disease varies widely. Based on testing in the general population and in defined groups for COVID-19, the percentage of asymptomatic COVID-19 positive individuals that do not develop symptoms ranges from 12.2 to 62.5% (14–18). In addition, asymptomatic infections of COVID-19 have been shown to be more common when the SARS-CoV-2 virus has a specific mutation, the 11083G>T mutation (19). Symptomatic, pre-symptomatic, asymptomatic individuals, and patients with COVID-19 can transmit COVID-19 to others (20–22).

There are similar viral loads at the start of infection among asymptomatic and symptomatic patients (7, 16) and viral load does not seem to correlate with disease severity as there is no statistical difference in viral load between asymptomatic and hospitalized patients with COVID-19 (23). Higher viral load in nasopharyngeal samples is more common in patients with an unfavorable outcome; however, a high viral load is not an independent risk factor for intensive care unit (ICU) admission or death (24).

The viable SARS-CoV-2 virus, cultured in Vero cells, can be isolated from the samples of patients with COVID-19 up to 24 days after symptom onset with more success in the earlier days (25–29). Viable SARS-CoV-2 virus cannot be isolated from all RT-PCR confirmed patients, as it is highly dependent on a high viral load (27). The likelihood of isolating a viable virus is significantly higher in the first week after the onset of symptoms than in the second and after 10 days, the probability drops to 6.0% (30). The virus is most commonly isolated from nasopharyngeal swabs and sputum but has also been isolated from saliva (25), endotracheal samples (28), stool (31), and urine (32). The viable virus has been cultured in samples from symptomatic, pre-symptomatic, asymptomatic, and re-detectable positive patients with COVID-19 (33–35).

The infectivity of SARS-CoV-2 that varies among patients with COVID-19 depends on multiple factors, such as their vaccination status. However, the highest viral load and infectivity are noted during the first 5 days of the symptomatic state.

## SARS-CoV-2 antibody response

### Seroconversion following SARS-CoV-2 exposure has been under intense investigation

Seroconversion (when antibodies against SARS-CoV-2 can be detected) has been reported to be at around day 12 after the onset of symptoms with some individual variability that is not associated with disease severity (36). The clinical significance of the individual isotype responses has been evaluated. Interestingly, higher immunoglobulin M (IgM), immunoglobulin M (IgG), and immunoglobulin M (IgA) SARS-CoV-2 specific antibodies have been associated with worse clinical outcomes (37–41). Patients with high levels of IgG and IgA anti-receptor-binding domain (RBD) antibodies were more likely to require hospital admission, mechanical ventilation, and fatal outcomes when compared to patients with lower levels. Non-hospitalized patients also had lower neutralizing antibodies (42). Patients with COVID-19 in the ICU had higher levels of IgA for RBD, S1, and N protein when compared to non-hospitalized patients (41).

It has become increasingly common to test for the presence of SARS-CoV-2-specific antibodies in the serum of individuals. It is performed for numerous reasons 1) to determine if an individual has been infected with the SARS-CoV-2 virus, 2) to determine the level of protection against re-infection, 3) to determine the level of protection in vaccinated individuals, and 4) to determine if an individual is contagious or not. However, it remains to be completely resolved if serological status against SARS-CoV-2 can be used to determine the non-infectious state. Excluding RT-PCR and measuring only antibodies for SARS-CoV-2 are not sufficient to indicate whether an individual has been infected in the first week of disease but after 21–35 days, the sensitivity of pooled IgG/IgM measurements rises to 96.0% (43).

The sensitivity and specificity of the various commercially available SARS-CoV-2-specific serologic assay kits vary, based on testing method and manufacturer. Currently, there are at least 222 commercialized SARS-CoV-2 antibody immunoassays that have received CE-*in vitro* diagnostic (IVD) certification (44).

### Immunological memory after COVID-19 and neutralizing antibodies

Immunological memory is formed after infection, but how long the memory lasts is highly dependent on the type of infection. Neutralizing antibodies are of special importance as they prevent the binding of the pathogen to the host cells. Studies have shown that antibodies formed after COVID-19 last at least 6 months or more in most patients, but the level of antibodies decreases with time (18, 45–47). Levels of IgG specific for the spike protein of SARS-CoV-2 are stable for over 6 months and the number of spike-specific memory B cells is higher 6 months after infection when compared to 1 month (46, 48).

The half-life of anti-spike protein IgG antibodies has been shown to be 184 days, with a shorter half-life for men (49). The spike protein consists of the S1 and S2 subunits, with the RBD situated within S1. The half-life of antibodies against different parts of the spike protein differs, with antibodies against S1 having the shortest half-life at 115 days, 125 days for antibodies against the RBD, and 344 days for antibodies against the S2 part (50).

The main neutralizing antibodies for SARS-CoV-2 have been found to be directed against the spike protein of the virus and the RBD domain, presumably, as they prevent respiratory epithelial cellular entry by the virus (51). Although the majority of today's known neutralizing antibodies disrupts angiotensin-converting enzyme 2 (ACE2) binding to the RBD, others have been found to recognize epitopes outside this site (52). The half-life of neutralizing antibodies against SARS-CoV-2 has been documented to be from 90 to 114 days (46, 53). Only 20.2% of the mean serum levels of SARS-CoV-2-specific antibodies in convalescent and vaccinated individuals is needed to confer a 50% protection against severe infection (53). Only time will tell how the antibodies for SARS-CoV-2 are maintained in the long term. However, it has been estimated that antibodies will maintain a 50% protection against COVID-19 infection for 1.5–2 years, while 50% protection against serious infection would last several years (49, 53). This can clearly represent a weakness in the efficacy of the vaccines, as the spike protein is prone to mutate, making the SARS-CoV-2 virus more infectious (54).

### Vaccine targets

After the SARS outbreak in 2003, studies reported neutralizing antibodies against the SARS-CoV spike protein (55). Since both SARS-CoV and SARS-CoV-2 utilize the attachment of spike protein to the human ACE2 receptor to invade host cells, it is crucial to develop neutralizing antibodies against the spike protein to induce protection against SARS-CoV-2 infection (56). The spike protein thus became the main target of vaccine development (52). Presently, Sputnik V, ChAdOx1, Spikevax, BNT162B2, Vidprevtyn, VLA2001, COVID-19 Vaccine Janssen, Nuvaxovid, and COVID-19 Vaccine from Sinovac all target the spike protein. This can clearly represent a weakness in the efficacy of the vaccines, as the spike protein is prone to mutate (57–59).

Recent results from a retrospective study based on the U.S. registry have shown that transfusion of plasma with high anti-SARS-CoV-2 antibody levels was associated with a lower risk of death when compared to plasma with lower antibody levels (60). In Iceland, Ronapreve^TM^ was used successfully in treating patients against the first variants of concern. However, with the emergence of the Omicron variant, the usefulness of many of the monoclonal antibody biologicals is dwindling. Sotrovimab^TM^ was effective against the BA.1 Omicron subvariant, but the effectiveness against the BA.2 Omicron subvariant is negligible.

Examining the neutralizing antibodies in seropositive and seronegative individuals after receiving the BNT162b2 mRNA (Pfizer) vaccine, it was seen that individuals who had been infected with SARS-CoV-2 produced antibodies with a higher neutralization potency and were less susceptible to escape variants of the virus. Suggesting that booster doses of the vaccine that induced a higher frequency of memory B cells are able to produce a broader range of neutralizing antibodies and target the escape variants (61). Moreover, neutralizing IgG and IgA antibodies have been detected in the breast milk of lactating women, reaching stable levels 14 days after the second dose (62). The third dose of BNT162b2 has also been shown to have a 93% effectiveness in preventing COVID-19-related hospital admission, 92% effectiveness in preventing against severe disease, and 81% effectiveness in preventing death, when compared to two doses administered at least 5 months before (63).

The declining efficacy of BNT162b2 in protecting against SARS-CoV-2 infections of the BNT162b2 vaccine has been reported 6 months after being fully vaccinated (two doses), the reason probably being due to waning immunity, rather than new variants, such as Delta. Importantly, however, the effectiveness of the vaccine in protecting against severe disease and hospital admissions did not wane (64). A fourth dose, administered 4 months after the third, has been shown to be efficacious against symptomatic disease. It did not show any substantial differences in humoral responses when compared to the third, suggesting that the third dose induced the maximal immunogenicity of the vaccine, whereas the fourth dose was able to restore the antibody levels (65).

The omicron variant harbors over 30 mutations in the coding region for the spike protein (66). Models, validated by experimental results, have suggested that the omicron variant is 2.8 times more contagious than the delta variant (67). This is of concern regarding the efficacy of the current vaccines targeting the spike protein of the SARS-CoV-2 virus. It has been shown that the efficacy of the BNT162b2 vaccine is still maintained, although at a lower level (68). One month (66, 69) after the second dose of the mRNA-1273 vaccine, the neutralization titers were 35 times lower against the omicron variant than the delta variant. While a booster dose of the vaccine increased the neutralization titer against the omicron variant 20-fold when compared to 1 month after the second dose of the vaccine (70). Three doses of mRNA COVID-19 vaccine have also been associated with protection against both the omicron and delta variants (65, 71), with the fourth dose able to restore the antibody levels comparable to the third dose, but not showing any difference in the levels of omicron-specific neutralizing antibodies (65).

Thus, it is clear that vaccines protect against infections with SARS-CoV-2 and even though the virus mutates, they still offer some protection against the new variants. However, vigilance is needed offering the science community a challenge to develop new vaccines tailored against new virus variants. Even though initial vaccines offer less protection against newer variants but they are still helpful as demonstrated by their ability to protect against severe disease, hospitalization, and death (72).

### Is it possible to determine non-infectivity?

A clear defining criterion when patients with COVID-19 cease to be infectious remains to be determined. This has a wide range of implications, such as public health recommendations, the safety of healthcare personnel, and international traveling to name a few.

Scientifically validated guidelines on this matter are required. Unfortunately, major differences exist between the current guidelines, and they are everchanging. The most common clinical and biological markers used to determine non-infectivity are viral RNA copies, viable viral isolation, symptom score, serology, and days from initial symptoms. Viral viability studies are the gold standard but are not practical for widespread use. Case studies where patients with COVID-19 have been followed have shown that infectivity can be maintained for a longer time than the 20-day transmission-based precautions recommended by the Centers for Disease Control and Prevention (CDC) (26, 73) or the 14–20 days of isolation as recommended by the European Center for Disease Prevention and Control (ECDC) for individuals with severe symptoms (74).

The quantification cycle (Cq, also known as threshold cycle (Ct)) value from the RT-PCR has also been used as a surrogate marker for infectivity, where Cq <35 is regarded as positive. Thus, a value of <20 has been shown to correlate with high viral load, whereas values of >35 might reflect a low contagious state with no detectable viable virus (27, 75). However, detection power is significantly affected by various factors (76), such as sampling location, swab technique, days from exposure, duration, and severity of symptoms among others. It is also important to note that one sample only gives a point estimate in time and the Cq values are most likely low (high viral load) before symptom onset and in the post-infectious state and as previously mentioned, re-infections with SARS-CoV-2 are common. However, a meta-analysis shows that a pooled estimate of how many people are re-detectable positive, in a cohort of recovered COVID-19 patients post discharge, is 14.8% and that the time from onset of symptoms to being re-detectable positive is 35.4 days (77). COVID-19 could therefore have a longer disease duration than previously thought.

Rapid antigen tests have significantly lower sensitivity and specificity than RT-PCR assays. These have been suggested as an alternative quick way to differentiate between contagious and non-contagious individuals (78, 79). In addition, in a comparative study of 122 CE-marked SARS-CoV-2 antigen rapid diagnostic tests (Ag RDTs), 78.6% met the authors' 75% sensitivity criteria in samples with relatively high viral load (Cq ≤ 25). Finally, only 20.8% met that criterion in samples with moderate viral load (Cq >25 to <30) and the majority were negative in samples with low viral load (Cq > 30). A Cochrane review on rapid antigen tests shows that these tests have a higher sensitivity for symptomatic patients with COVID-19 than asymptomatic (72.0 vs. 58.1%, respectively) and in patients that have Cq ≤ 25 than in those with higher Cq (94.5 vs. 40.7%, respectively). In addition, in the first week of symptoms, the average sensitivity of the rapid antigen tests was higher than in the second week (78.3 vs. 51.0%, respectively) (78). Another recent study found that the rate of false negative rapid antigen testing was noted in 87 of the 807 tests. Furthermore, the negative predictive value correlated strongly with the time of symptoms with a negative predictive value of rapid antigen testing being 80–100% for symptoms lasting <5 days, whereas, the negative predictive value for the longer duration was only 50% (80). Thus, since very high viral load most often coincides with a symptomatic state, it is clear that rapid antigen tests are, at best, only useful in identifying potentially infected individuals with symptoms highly suggestive of COVID-19 that should be corroborated with PCR testing (81). It has been shown that if viral load fell below 10^6^ copies/ml in patients with COVID-19, it was not possible to culture viable virus despite positive RT-PCR up to day 28 (27). In addition, in an earlier report, it was suggested that patients with Cq above 33–34 by RT-PCR technology might not be contagious and might be used to determine if they could be safely discharged or relieved from strict confinement. Similar findings have been observed by others suggesting that Cq values above 30–33 might be used to define the viability of replicating the SARS-CoV-2 virus (1). In addition, comparing Cq values between laboratories can be problematic, as large variation has been found with a quantitative comparison between samples (82). Where the method of sampling and sampling location are of importance, with the nasopharyngeal swabs still being the gold standard while throat swabs are not recommended due to the low sensitivity and positive predictive value (83). Thus, based upon these and similar findings, this has been further stratified into the following groups of viral load: high (Cq 17–25), moderate (Cq 25–30), and low (Cq 30–36).

**Figure 1:**
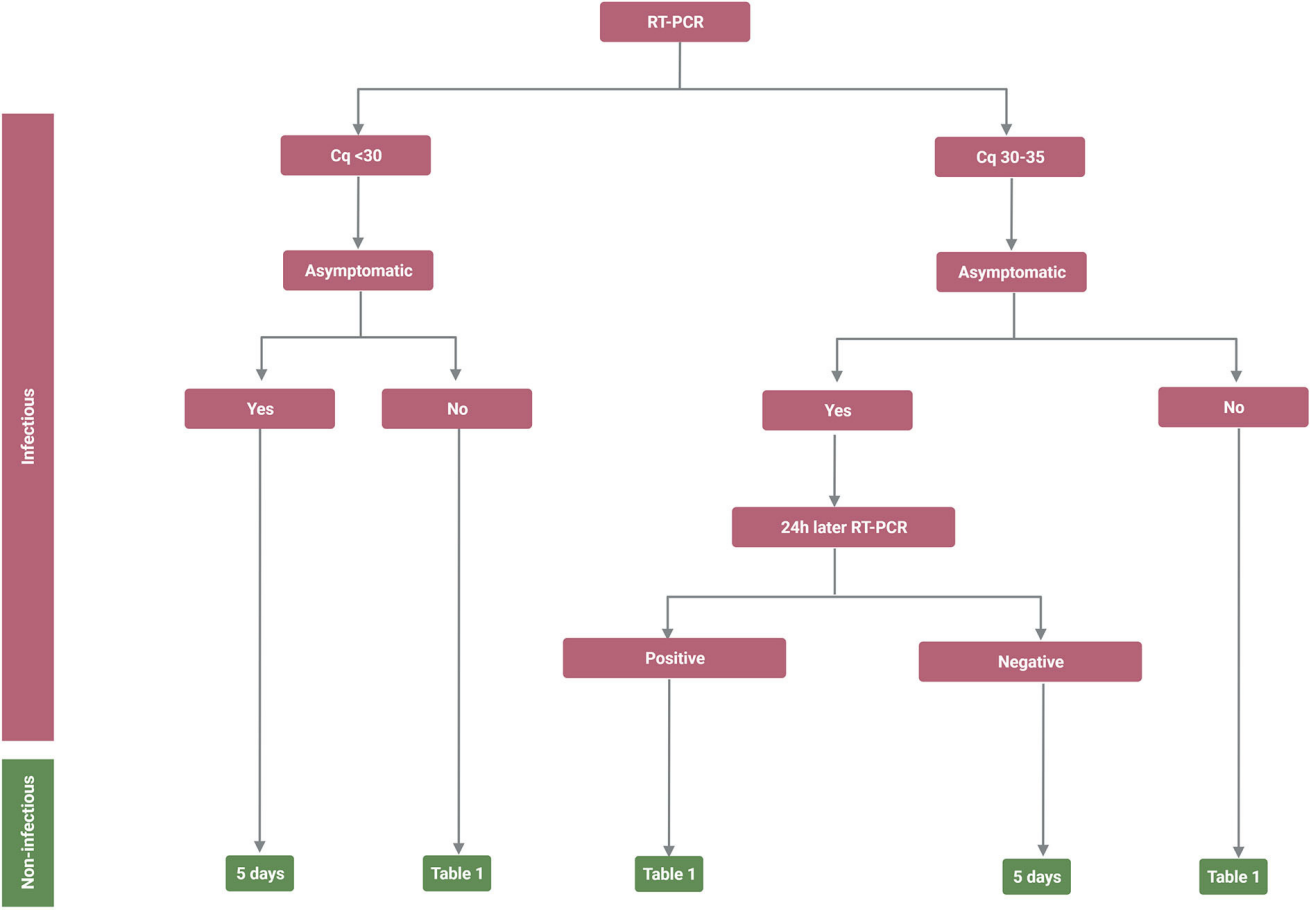
Infectivity and non-infectivity of fully vaccinated, immune competent, patients infected with Severe Acute Respiratory Syndrome Coronavirus 2 (SARS-CoV-2). Flow chart depicting the suggested strategy on how to isolate SARS-CoV-2 infected individuals to minimize the likelihood of releasing infectious individuals back into society. The flow chart takes into account that it is the last symptom or reverse transcription polymerase chain reaction (RT-PCR) measurement (Cq value) that defines in what category the individual will place since patients will be in different stages of the disease when they are sampled. *It should be noted that the positive predicted value of the antigen test can vary and it may therefore be important to confirm the diagnosis with RT-PCR. Created with BioRender.com.

Based on the data discussed above and the clinical experience of the physicians managing the pandemic in Iceland, a flow chart (Figure 1, Table 1) was created, proposing a strategy on how to determine a low risk for viral transmission (non-infectivity) in fully vaccinated individuals based upon their symptomatic state. A similar strategy has already been proven to be highly successful at Landspitali University Hospital in Reykjavik, Iceland.

**Table 1:** Determination of non-infectivity in relation to vaccination status and symptoms.

**Vaccination status**	**Symptoms**	**Days of isolation until non-infectivy**
Three doses^a^	No or mild symtpoms	5 days, no need for second PCR or antibody measurements
Two doses^b^	No symptoms or low symtpoms	7 days, no need for second PCR or antibody measurements
Partially and unvaccinated^c^		10 days, no need for second PCR or antibody measurements.
Serious COVID-19 disease, indipendent of vaccionation status	Serious COVID-19 symptoms needing dexamethasone, tocilizumab, ICU admission, respirator.	14 days and patient has N-protein specific antibodies and/or 1 negative PCR test OR 21 days after diagonsis

a *Three doses of vaccine or two doses of vaccine and recovered from COVID-19*.

b *Two doses of vaccine or one dose of vaccine and recovered from COVID-19*.

c *No vaccination or only one dose*.

## Concluding remarks

Numerous attempts have been made to provide evidence-based protocols to establish non-infectivity, particularly for determining when to stop quarantine of infected individuals, when healthcare workers can return to work, and more importantly for infected immunocompromised individuals.

While rapid antigen tests do not have the sensitivity or specificity of RT-PCR tests, they can contribute to the removal of asymptomatically infected or pre-symptomatic SARS-CoV-2 spreading individuals from the general population. In addition, in selected cases in asymptomatic individuals, in-depth SARS-CoV-2-specific IgM/IgG/IgA levels might provide a better overview of the individuals' timeline of infectivity.

Thus, the suggested flow chart will hopefully provide some insight into how to minimize the likelihood of releasing an infected and contagious individual from all restrictions either within the hospital or within general public settings.

## Author contributions

All authors listed have made a substantial, direct, and intellectual contribution to the work and approved it for publication.

## Conflict of interest

The authors declare that the research was conducted in the absence of any commercial or financial relationships that could be construed as a potential conflict of interest.

## Publisher's note

All claims expressed in this article are solely those of the authors and do not necessarily represent those of their affiliated organizations, or those of the publisher, the editors and the reviewers. Any product that may be evaluated in this article, or claim that may be made by its manufacturer, is not guaranteed or endorsed by the publisher.
